# 

**Published:** 1959-01

**Authors:** 


### THE
### MEDICAL JOURNAL
### OF THE
### SOUTH-WEST

*Incorporating*

*The Bristol Medico-Chirurgical Journal*

PUBLISHED UNDER THE AUSPICES OF THE

BRISTOL MEDICO-CHIRURGICAL SOCIETY

*1959 Vol. 74*

J. W. ARROWSMITH LTD., WINTERSTOKE ROAD, BRISTOL

W1
ME359
v. 74-76
1959-61
c.1

### *1959 Contents to Vol. 74*

*page*

The Elimination of Piltdown Man. By Hanbury Hazell, L.D.S.R.C.S. . . . 1

Recent Developments in Cardiac Surgery. By R. E. Horton, M.B.E., M.S., F.R.C.S. and J. Clutton-Brock, F.F.A.R.C.S.. . . . . . . 6

Recent Work on the Demyelinating Diseases. By N. S. Alcock, M.D., F.R.C.P. . 17

Prolonged Coma in Barbiturate Poisoning. By S. G. Flavell Matts, M.B. (B'ham), M.R.C.S., M.R.C.P. (Ed.). . . . . . . . 20

Harpes Zoster with Chicken-pox and Lymphosarcoma. (Clinical Pathological Conference) . . . . . . . . 22

A Review of Acute Intestinal Obstruction. By P. J. W. Monks . . . 31

Squint. By Calbert I. Phillips . . . . . . 40

Transporting Severely Paralysed Patients. By James Macrae, M.D. . . 45

Aplastic Anaemia with Post-Transfusional Haemosiderosis (Clinical Pathological Conference) . . . . . . . . 47

Decay of the Teeth. By A. I. Darling, D.D.SC., M.D., F.D.S.R.C.S., L.R.C.P., M.R.C.P. . . . . . . . . . 53

Lung Cancer—A Symposium. By J. E. G. Pearson, M.D., F.R.C.P. . . 61

Alveolar Cell Carcinoma of Lung. (Clinical Pathological Conference) . . 70

Baby Farming in England. By Philip Evans, M.D., M.SC., F.R.C.P. . . 83

The Use of an Animal Lung as Oxygenator in a Heart-Lung Machine. By M. G. Wilson, CH.M., F.R.C.S. and others. . . . . . . 90

Notes and News . . . . . . . 27, 51, 77

Appointments . . . . . . . 27, 51, 78

News from Societies . . . . . . . . . 74

3375K

### THE MEDICAL JOURNAL OF THE SOUTH-WEST

*(Incorporating the Bristol Medico-Chirurgical Journal)*

Established 1883

VOL. 74 (i) January 1959 NO. 271

### Contents

| | | Page |
|---|---|---|
| THE ELIMINATION OF PILTDOWN MAN | *Mr. Hanbury Hazell* | 1 |
| RECENT DEVELOPMENTS IN CARDIAC SURGERY | *Mr. R. E. Horton and Dr. J. Clutton-Brock* | 6 |
| RECENT WORK ON THE DEMYELINATING DISEASES | *Dr. N. S. Alcock* | 17 |
| PROLONGED COMA IN BARBITURATE POISONING | *Dr. S. G. Flavell Matts and Dr. H. G. Handel* | 20 |
| HERPES ZOSTER WITH CHICKEN-POX AND LYMPHOSARCOMA | *Clinical Pathological Conference* | 22 |
| NOTES AND NEWS . . . . . . . . | | 27 |
| APPOINTMENTS . . . . . . . . | | 27 |

*PUBLISHED QUARTERLY*

*ANNUAL SUBSCRIPTION* **16/-** *POST FREE*

PUBLISHED UNDER THE AUSPICES OF THE
BRISTOL MEDICO-CHIRURGICAL SOCIETY

### *Editorial Committee*

Dr. J. E. CATES, Department of Medicine, Bristol Royal Infirmary. *Editor.*
Dr. N. J. BROWN, Southmead Hospital, Bristol. *Assistant Editor.*
Dr. J. MACRAE, Ham Green Hospital.
Dr. R. C. WOFINDEN, Central Clinic, Bristol.
Mr. A. E. S. ROBERTS, Medical Library, University of Bristol. *Secretary.*

### *Local Correspondents*

*Bath* . . Mr. A. D. BATEMAN, 3 The Circus, Bath.
*Exeter* . . Dr. L. N. JACKSON, Mount Jocelyn, Crediton, Devon.
*Gloucester* . Dr. R. F. JARRETT, 9 College Green, Gloucester.
*Plymouth* . Dr. C. R. CROFT, Lexden, Hartley Av., Plymouth.
*Taunton* . Dr. M. MCALLEY, 1 The Crescent, Taunton.
*Torquay* . Dr. A. ROBINSON THOMAS, 20 Devon Square, Newton Abbot, Devon.
*Truro* . . Dr. F. D. M. HOCKING, St. Andrews, Perranporth, Cornwall.
*Yeovil* . . Mr. J. A. VERE NICOLL, Knapp House, Yeovil, Somerset.

### NOTICE TO CONTRIBUTORS

Original articles are invited, provided they have not been published elsewhere. Notes of forthcoming events, meetings held, degrees conferred, staff appointments, and other local news are welcomed. The editorial committee reserves the right to make literary corrections.

Illustrations should always be supplied ready for reproduction. All re-touchings or other operations required will be charged to the author. Line drawings should be drawn in Indian ink. We regret that we must ask authors to pay for making blocks, if this is necessary.

J. W. ARROWSMITH LTD., WINTERSTOKE ROAD,
BRISTOL.

### Notes and News

*OBITUARY*

A. T. Todd.

### WEST OF ENGLAND CHILD HEALTH GROUP

*Secretary:* Dr. P. G. Roads, Central Health Clinic, Tower Hill, Bristol 2.

Details of the May and June meetings are as follows:

5th May, at 5.30 p.m., at the Children's Hospital: Miss Cecile Hopkins, M.R.C.S., L.R.C.P., D.P.H., **"Problem Families".**

25th June, at 6 p.m., at Manor Hall, Clifton: Mrs. Marjorie Tait, B.A., PH.D., **"The Care of Young Ladies".**

### EXAMINATION RESULT

*M.D. (Aberdeen)*: R. S. Crow (with commendation).

### UNIVERSITY OF BRISTOL

*M.B., Ch.B.*

Pass

Chapman, B. A. E.
Clark, M. B.
Clay, J. D.
Cormack, V. R.
Henley, G. D.
Hughes, V. C.
Lassey, H. S.
Mapstone, R.
McClements, B. R. S.
Ogden, A. H.
Parker, R. K. H.
Power, P. C.
Shield, J. E. H.
Shute, G. S.
Simon, A. P.
Webster, L. F.

### PRIZES

*British Dental Association Prize in Dental Anatomy:* D. Hill.
*George Fawn Prize:* I. M. Vickery.

### Appointments

E. W. Bradford, D.D.SC., senior lecturer in preventive dentistry and parodontal diseases at St. Andrews, has been appointed to the chair of dental surgery in the University of Bristol. Professor A. I. Darling will assume the title of "professor of dental medicine".

K. W. Aron, M.B. (Bristol), D.P.M., consultant child psychiatrist to develop child guidance services, Glamorgan.

W. Ross Ashby, M.A., M.D., director of research at Barnwood House, Gloucester, has been appointed director of the Burden Neurological Institute, Bristol, in succession to Professor F. L. Golla, who is retiring.

Dorothy M. Boisen, M.B. (Bristol), medical officer, School Health Service, Birmingham.

P. O. Nicholas, M.B. (Bristol), D.C.H., Assistant M.O. and School M.O., Castleford, Yorkshire.

P. R. H. Slade, Ch.M. (Bristol), F.R.C.S., senior registrar in thoracic surgery, Papworth Hospital.

### *UNITED BRISTOL HOSPITALS*

*SEPTEMBER TO NOVEMBER, 1958*

| *Name* | *Appointment* | *Formerly* |
|---|---|---|
| RAPER, A. B., B.SC., M.D., M.R.C.P., D.T.M. & H. | Consultant Pathologist (specializing in Haematology). | Member of the senior Scientific Staff, National Institute of Medical Research and previously Senior Pathologist, Uganda Medical Service. |

| *Name* | *Appointment* | *Formerly* |
|---|---|---|
| DAVIS, R. REED, F.R.C.S., F.R.C.S.E. | Registrar in Orthopaedics. | Chief Assistant to Clinical Di[illegible]tor, Birmingham Accide[illegible] Hospital. |
| MARTYN, SHEILA A., M.B., CH.B., (B'tol.), M.R.C.O.G. | Registrar in Obstetrics and Gynaecology. | Locum Registrar, Southm[illegible] Hospital (formerly Tempo[illegible] Lecturer, University [illegible] Leeds). |
| BURNS, D. M. J., M.B., B.CHIR., (Camb.), M.R.C.S., L.R.C.P. | Registrar in Ophthalmology. | S.H.O., General Surgery, N[illegible]castle General Hospital. |
| MAYO, K. M., M.B., CH.B., D.M.R.D. | Registrar in Radiology (Diagnostic). | Research Assistant. |
| HOUGHTON, E. A. W., M.B., CH.B. (B'tol.), D.M.R.D. | Registrar in Radiology (Diagnostic). | Part-time Registrar in Ra[illegible]diagnosis., U.B.H. |
| WILLIAMS, D. P. C., M.B., CH.B. (L'pool), D.L.O. | Registrar in Ear, Nose and Throat Surgery. | Civilian Ear, Nose and Th[illegible] Specialist of Central Me[illegible] Establishment, R.A.F. |
| PHILPOTT, B., M.B., B.S., (Lond.), L.R.C.P., M.R.C.S., D.A. | Registrar in Anaesthetics. | S.H.O. in Anaesthetics, Cha[illegible] Cross Hospital. |
| JENKINS, D. G., F.R.C.S. | Locum tenens Senior Registrar in Genito-Urinary Surgery. | — |

### *SOUTH WESTERN REGIONAL HOSPITAL BOARD*

### *SEPTEMBER TO NOVEMBER* 1958

### *BRISTOL*

| *Name* | *Appointment* | *Formerly* |
|---|---|---|
| SLADE, N., M.B., CH.B. (B'tol.), F.R.C.S. | Consultant Genito-Urinary Surgeon, Bristol Clinical Area. | Senior Registrar to Depart[illegible] of Urology, U.B.H. & So[illegible]mead Hospital. |
| HANKS, G. A., M.B., CH.B. (B'tol.), D.R.C.O.G. | Surgical Registrar, Weston-super-Mare General Hospital. | S.H.O. in General Sur[illegible] B.R.I. |
| TALWAR, D. R., M.B., B.S., D.A. (Bombay). | Anaesthetic Registrar, Southmead Hospital, Bristol. | Anaesthetic Registrar, De[illegible]shire Royal Infirmary. |

### *NORTH GLOUCESTERSHIRE*

| *Name* | *Appointment* | *Formerly* |
|---|---|---|
| HAMILTON, A. F., L.D.S., R.C.S., H.D.D., R.C.S. (Edin.), F.D.S., R.C.S. (Eng.). | Consultant Dental Surgeon, North Gloucestershire Clinical Area. | Assistant Dental Surgeon, Pl[illegible] Surgery & Jaw Injuries Ce[illegible] Stoke Mandeville Hosp[illegible] |
| CROUCH, H. E., M.B., B.CH., B.A.O. (Belfast). | Clinical Assistant in Dermatology, Stroud Hospital and Gloucester Royal Hospital, Gloucester. | — |
| MCCARTHY, T. F., B.SC., M.D. (Wales), M.R.C.P. (Edin.). | Assistant Geriatrician, North Gloucestershire Clinical Area. | Resident Physician, Nab [illegible] Sanatorium, Marple. |
| SHEPHARD, G. D. H., M.B., B.CHIR. (CANTAB). D.OBST., R.C.O.G. | G.P. Surgeon, Tewkesbury Hospital. | Is in general practice in Tew[illegible]bury. |

### BATH

| Name | Appointment | Formerly |
|---|---|---|
| VAN DEN BRUL, P. J., M.B., B.S. (LOND.). | Anaesthetic Registrar, Bath Group of Hospitals. | Resident Anaesthetist, R.U.H., Bath. |
| STEWART, D. J., M.B., CH.B. (GLASGOW). | Re-appointed for further year as Clinical Assistant in Orthopaedic & Traumatic Surgery, R.U.H., Bath. | Is in general practice in Bath. |
| WATERS, M. A., M.B., B.S. (LOND.), M.R.C.P. (EDIN.), D.P.M. (CONJOINT). | Consultant Psychiatrist, Roundway Hospital, Devizes. | Assistant Psychiatrist, Horton Hospital, Epsom. |

### SOUTH SOMERSET

| Name | Appointment | Formerly |
|---|---|---|
| WEBB, B. W., M.D. (LOND.), M.R.C.P. (LOND.), D.C.H. (ENG.). | Consultant Paediatrician, South Somerset Clinical Area. | Senior Registrar, Children's Department, King's College Hospital & Belgrave Hospital for Children. |
| ISAAC, D. H., M.D. (LOND.), M.R.C.P. (LOND.). | Consultant Physician, South Somerset Clinical Area. | Senior Medical Registrar, U.B.H. |

### DEVON AND CORNWALL

| Name | Appointment | Formerly |
|---|---|---|
| MCCORMICK, A. J. A., M.B., CH.B. (ED.), F.R.C.S. (ED.), D.O.M.S. | Ophthalmologist, Exeter Clinical Area (Torquay). | Senior Registrar in Ophthalmology, U.B.H. |
| ELLIS, D. D., M.B., B.CH., B.A.O. (BELFAST), D.P.M. (R.C.P.S.I.). | Consultant Psychiatrist, St. Lawrence's Hospital, Bodmin. | Assistant Psychiatrist, Winwick Hospital Warrington. |
| STIRK, D. I., F.R.C.S. (ENG.). | Consultant Surgeon, Exeter Clinical Area (North Devon Infirmary, Barnstaple). | Senior Surgical Registrar, Singapore General Hospital. |
| GARDNER, A. M. N., B.M., B.CH. (OXON.), F.R.C.S. (ENG.). | Consultant Surgeon, Exeter Clinical Area (Torquay). | Senior Surgical Registrar, Radcliffe Infirmary, Oxford & Northampton General Hpl. |
| WALKER, H. M., M.B., B.S. (MELBOURNE). | Registrar in Obstetrics & Gynae., Royal Devon & Exeter Hospital (appt. with Southmead Hospital). | S.H.O. in Obstetrics & Gynae. (joint St. James's Hospital, Balham). |
| NEWTON, A. J., M.B., B.S. (LOND.). | Registrar in Orthopaedic and Traumatic Surgery to work at Torbay Hospital, Torquay and Princess Elizabeth Orthopaedic Hospital, Exeter, on an exchange basis. | Locum S.H.O. to Fracture & Accident Department, Gloucestershire Royal Hospital, Gloucester. |
| PARKES, J., M.A., M.B., B.CHIR. (CANTAB.), M.R.C.P. (EDIN.). | Re-appointed for further year as Clinical Assistant in Paediatrics, Torquay, | Is in general practice in Torquay. |
| WILLIAMSON, F., M.D. (TORONTO). | Surgical Registrar, Torbay Hospital, Torquay and Newton Abbot Hospital. | Locum Surgical Registrar, Maidenhead General Hospital. |
| DUNCAN, H. LEE, M.B., CH.B. (EDIN.), F.R.C.S. (EDIN.). | Clinical Assistant in Surgery, West Cornwall Hospital, Penzance. | Is in general practice in Penzance. |

